# Improved data validity in the Swedish Register of Palliative Care

**DOI:** 10.1371/journal.pone.0186804

**Published:** 2017-10-19

**Authors:** Lisa Martinsson, Per-Anders Heedman, Staffan Lundström, Bertil Axelsson

**Affiliations:** 1 Department of Radiation Sciences, Umeå University, Umeå, Sweden; 2 Palliative Education and Research Center in the County of Östergötland, Vrinnevi Hospital, Norrköping, Sweden; 3 Department of Palliative Medicine, Stockholms Sjukhem Foundation, Stockholm, Sweden; 4 Department of Oncology-Pathology, Karolinska Institutet, Stockholm, Sweden; 5 Department of Radiation Sciences, Unit of Clinical Research Centre–Östersund, Umeå University, Umeå, Sweden; International University of Health and Welfare School of Medicine, JAPAN

## Abstract

**Introduction:**

The Swedish Register of Palliative Care (SRPC) is a national quality register that collects data about end-of-life care from healthcare providers that care for dying patients. Data are used for quality control and research. Data are mainly collected with an end-of-life questionnaire (ELQ), which is completed by healthcare staff after the death of a patient. A previous validity assessment of the ELQ showed insufficient validity in some items including symptom relief. The aim of this study was to examine the validity of the revised ELQ.

**Materials and methods:**

Data from 100 consecutive patients’ medical records at two specialised palliative care units were used to complete new ELQs, which were then compared to the ELQ registrations from the SRPC for the same patients. The level of agreement was calculated for each ELQ item. To account for the possibility of the agreement occurring by chance, Cohen’s kappa was calculated for suitable items. To examine the extent of registration mistakes when transferring the paper form to the web, the original paper versions of the ELQ filled out at the units were compared to data from the ELQs reported to the SRPC.

**Results:**

Level of agreement between ELQ registrations from the SRPC and the new ELQs based on the medical records varied between 0.55 and 1.00, where 24 items showed level of agreement above 0.80 and 9 items showed level of agreement below 0.80. Cohen’s kappa with 95% confidence intervals was calculated for 24 items. The kappa values showed that two items had poor agreement, four fair agreement, 11 moderate agreement, five good agreement and two very good agreement. The level of agreement varied between 0.93 and 1.00 when comparing the ELQ registrations in the SRPC and the paper forms.

**Conclusion:**

The revised ELQ contains more items with high levels of agreement between registrations in the SRPC and notes in the patients’ medical records when compared to the previous version. Validating issues around symptom assessment remains a challenge in our model of quality assessment.

## Introduction

The Swedish Register of Palliative Care (SRPC) is a national quality register founded in 2005 with an aim to evaluate and improve end-of-life care for dying patients in Sweden, regardless of diagnosis, place of residence and level of care [1]. Data from the SRPC provide unique information about Swedish end-of-life care. Data are used by the healthcare institutions for quality control, and have also been used in several clinical research projects [2–9]. In our experience, the SRPC has been generally accepted as a tool for assessment of care quality by the specialised palliative care as well as in nursing homes and hospitals. The yearly proportion of deaths in Sweden reported to the SRPC has increased from 5% to 68% during 2006–2014 [10,11]. In 2015, 60,013 patients were reported to the SRPC, which corresponds to 66% of all deaths in Sweden. The completeness of the SRPC is even higher for patients with cancer: in 2015 87% of all patients that died from cancer in Sweden were reported to the SRPC [12]. The development of the SRPC has been described by Lundström et al. [1].

The end-of-life questionnaire (ELQ) is the main source of register data in the SRPC, and its ability to generate accurate data is central to the register purposes. The ELQ is completed by healthcare staff after the death of a patient and focuses on the last week of life. Registration in the SRPC is web-based, but some units first complete a paper version of the ELQ which is later used to complete the web form [1]. All questions have to be answered in the web form before submission, which results in no missing data [13]. Stored data are matched with the central population register to ensure that only people that are deceased are included [1].

Items in the ELQ are used to calculate national palliative quality indicators defined by the Swedish National Board of Health and Welfare [1]. A quality indicator is defined as an explicitly defined and agreed upon measurable item that gives an indication of the quality of care, referring to clinical outcomes, processes (for example, following of guidelines) or structures [14–16]. Many quality indicators in palliative care are focused on specific populations, such as patients with incurable cancer, and on specific types of treatment [14,15], while the SRPC collects data regardless of diagnosis, place of death or level of care [1].

When the first version of the ELQ was developed, no similar questionnaire was found in the literature. It was thus developed without a previous model and was based on the core values of high quality end-of-life care proposed by the British Geriatrics Society [17]and on clinical experience. The first version of the ELQ was in use from January 2005 to March 2007. In May 2007 some linguistic clarifications of the ELQ were done, and some questions were added [18]. During 2009 Martinsson et al. examined the validity of the ELQ and found that some items in the questionnaire were not sufficiently valid, including the items about symptom relief. Eight of the items in the questionnaire fell below 0.80, which was the preset limit for acceptable level of agreement between data reported to the register and data found in the patients’ medical records [13]. A similar validation study, outside the arena of specialised palliative care, was planned by Gholiha et al., but the study could not be conducted as planned because of lack of documentation in medical records [19].

During 2010, the ELQ was modified based on the results from Martinsson et al. [13] and Gholiha et al. [19] and on comments left by healthcare staff who had used the questionnaire [20]. In January 2011 an updated version of the ELQ was launched. In that version, more items were assessed (for example, a question about assessment of oral care was added) and there was a larger emphasis on documentation, thus focusing more on processes and less on outcomes of care. In March 2012 a question about whether there was a documented decision by a physician to shift treatment and care to end-of-life care was added [21]. English translations of the previous and the revised versions of the ELQ are found in the supplementary material.

### Aim

The aim of this study was to assess the validity of the revised version in use from January 2011 of the end-of-life questionnaire from the SRPC.

### Ethics

This study was approved by the Regional Ethical Review Board in Linköping, Sweden (registration number 2012/152-31). Patients included in the study were deceased and had already been registered in the Swedish Register of Palliative Care by the patient responsible nurse or physician. The working procedure and study design was approved by the local ethics committee and no informed consent was required from next of kin.

## Materials and methods

### Planning and setting

Data were collected from the units of specialised palliative home care and the palliative inpatient ward at the Linköping University Hospital, Sweden. These units have a systematic and structured way of documenting important aspects of end-of-life care in computerised medical record modules included in the patients’ regular medical records. There are three modules: one used for the occasion when a physician documents a decision to shift treatment and care to end-of-life care, one for daily assessments after that decision, and one for notes regarding the death of the patient. These modules include items similar to the items in the ELQ. According to local routines at these palliative care units, after the death of a patient the staff involved in the end-of-life care complete a paper version of the ELQ, which is later transferred to the web-based form by a secretary.

Sample size was set to 100 patients. Inclusion criteria required that the patient had died either with support from the specialised palliative home care team or at the palliative ward, that a documented decision to shift treatment and care to end-of-life care had been included in the patient’s end-of-life module, and that a paper version of the ELQ had been completed and reported to the SRPC. To be able to examine the extent of registration mistakes when transferring the paper form to the web, only patients where a completed paper version of the ELQ could be found were included.

### Data collection

Deceased patients were identified through the units’ own monitoring routines, using their personal identification numbers. Data from 177 consecutive patients who had died between January and September 2012 were examined. Information from the end-of-life modules and other medical record notes about the patients’ end-of-life care was used to complete new ELQs for the included patients. The researcher who collected data and completed these ELQs was blinded to the ELQs previously completed by staff.

### Data analysis

Data from the ELQs completed by the researcher were compared with data from the ELQs reported to the SRPC by the units. The patients were identified in the register data by their personal identity numbers (question 2). Data generated from question 27 (about team satisfaction) are not used for scientific purposes by the SRPC and were not included in the analysis. Questions 28 (date the ELQ was completed), 29 (whether the ELQ was completed by a single employee or jointly by staff) and 30 (name and profession of registrant) were not analysed, since no data about these were found in the medical records. In question 7 (about causes of death), only the answering alternatives “Cancer”, “Cardiovascular disease” and “Pulmonary disease” were examined, since the other causes of death listed in this question were very uncommon in the study population.

Only cases where there was a legit answer available in both the SRPC data and the new ELQs based on the medical records were used when analysing each item. The medical records were considered as golden standard and thus items that could not be answered for all cases because of lacking documentation in their medical records were excluded. Some ELQ items are follow-up questions that were not to be answered for all cases, depending on the answer to the previous question. Such items where less than 50 cases generated answers are not reported, since we estimated that this would make the reported proportion too dependent on pure chance. When comparing the new ELQs based on medical records and the ELQs reported by the units in questions 13a, 14a, 15a, 15b, 18, 20a1, 20b1, 20c1, 20d1, 20e1, 20f1, 22, 23 and 26, the answering alternatives “No” and “Don’t know” were merged together in the analysis. However, they were not merged when comparing the paper forms of the original ELQ and the ELQs reported online by the units.

The level of agreement between ELQs completed by the researcher and the ELQs reported to the SRPC by the units was calculated using SPSS. ELQ items with a level of agreement below 0.80 were identified. To account for the possibility of the agreement occurring by chance, Cohen’s kappa with 95% confidence interval was calculated for the suitable categorical ELQ non-multiple choice items. The kappa values were used to categorise the agreement of the items based on the limits proposed by Altman: <0.20 as poor agreement, 0.20–0.39 as fair, 0.40–0.59 as moderate, 0.60–0.79 as good and 0.80–1.00 as very good agreement.[22]

To examine the extent of registration mistakes when transferring the paper form to the web, the original paper versions of the ELQ filled out at the units were compared to data from the ELQs reported to the SRPC. The levels of agreement were calculated, and Cohen’s kappa coefficient with 95% confidence intervals was calculated for the same items as in the main analysis.

## Results

Out of the 177 patients examined, 77 were excluded (Table 1) and 100 remained (Table 2).

**Table 1:** Number of excluded patients and reasons for exclusion.

Reason for exclusion	Excluded patients (n)
No documented decision to shift treatment and care to end-of-life care using the end-of-life module	23
No completed paper version of the ELQ found	53
Not reported to the SRPC despite a paper version of the ELQ found at the unit	1
Total of excluded patients	77

**Table 2:** Gender, age, place of death and cause(s) of death for the included 100 patients, according to their medical records (some patients had multiple causes of death).

Gender (n)	Women	52
	Men	48
Age (years)	Median	72.5
	Range	34 to 100
Cause(s) of death (n)	Cancer	88
	Cardiovascular disease	16
	Pulmonary disease	12
Place of death (n)	Own home with support from the palliative home care team	55
	Palliative inpatient ward	41
	Nursing home with support from the palliative home care team	3
	Hospital ward without palliative specialisation, with support from the palliative home care team	1

### Comparison between ELQ registrations from the SRPC and new ELQs based on the medical records

The six items about symptom relief (20a2, 20b2, 20c2, 20d2, 20e2 and 20f2) were excluded from the analysis because there was not enough information in the medical records to answer the questions for all study patients (Table 3). Despite this being a specialised palliative unit, evaluation of pain medication was not documented in the medical records in 45 cases. After exclusion of the items about symptom relief, four ELQ items that were follow-up questions only to be answered when certain answer alternatives were chosen in the previous question remained. Two of these (number 13b, 14b) were excluded from further analysis because less than 50 cases remained. In the other two (15b, 15c), 81 respectively 79 cases were used for analysis (Table 3).

**Table 3:** Number of valid cases for the ELQ items where cases were missing in the comparison between ELQ registrations from the SRPC and new ELQs based on the medical records for the individual ELQ items.

Question in ELQ where all 100 cases were not analysed	Valid cases (n)	Reason for cases missing	
		Follow-up question not to be answered because of the answer to the previous question (n)	Not enough information in the medical records to answer the question (n)
13b. Documentation of pressure ulcer at arrival	8	92	-
14b. Documented pressure ulcer at death	24	76	-
15b. Disorder noted during oral health assessment	81	19	-
15c. Documented assessment of oral health *	79	21	-
20a2 Pain relief	21	34	45
20b2 Death rattle relief	20	55	25
20c2 Nausea relief	4	92	4
20d2 Anxiety relief	8	62	30
20e2 Dyspnoea relief	4	81	15
20f2 Confusion relief	1	78	21

* Item used to calculate quality indicator.

**Fig 1:**
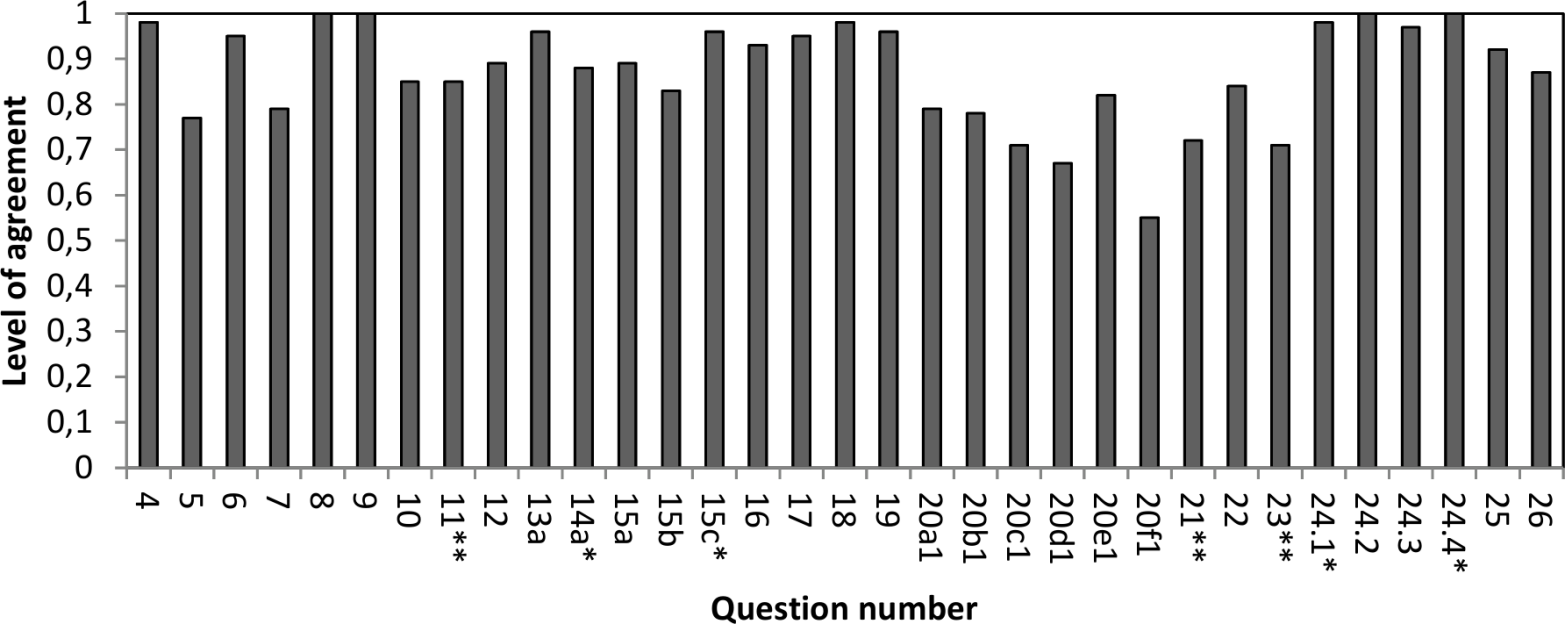
Level of agreement for 33 ELQ items. * Items used to calculate quality indicators. ** Items used to calculate quality indicators under development.

Level of agreement between ELQ registrations from the SRPC and the new ELQs based on the medical records varied between 0.55 and 1.00, where 24 items showed level of agreement above 0.80. The level of agreement fell below 0.80 for 9 items (Fig 1).

**Fig 2:**
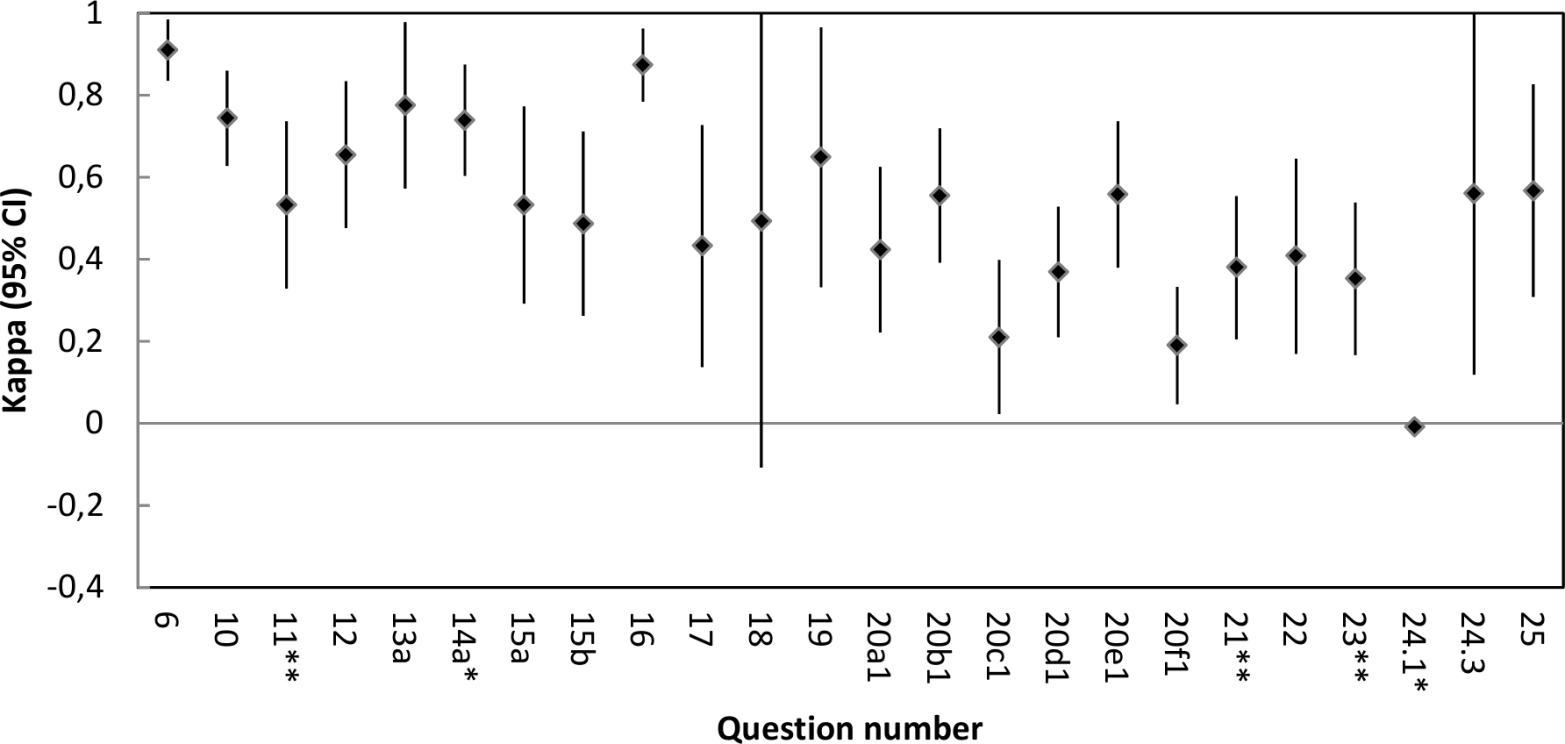
Cohen’s kappa values and 95% confidence intervals for 24 ELQ items. * Items used to calculate quality indicators. ** Items used to calculate quality indicators under development.

In six categorical questions (8, 9, 15c, 20f.2, 24.2 and 24.4) only one answering alternative had been used, and thus Cohen’s kappa could not be calculated. Cohen’s kappa with 95% confidence intervals was calculated for 24 items. The kappa values showed that two items had poor agreement, four fair agreement, 11 moderate agreement, five good agreement and two very good agreement. One question had kappa below 0, and in one question the lower bound of the 95% confidence interval fell below 0, indicating that the results were no better than chance (Fig 2).

All of the four ELQ items (question 14a, 15c, 24.1 and 24.4) used by the Swedish National Board of Health and Welfare to calculate quality indicators showed high levels of agreement (between 0.88 and 1.00) (Fig 1), but Cohen’s kappa could not be calculated for question 15c and 24.4. Question 24.1 and 14a had poor respectively good agreement according to their kappa values (Fig 2). Three items (question 11, 21 and 23) are used by the Swedish National Board of Health and Welfare to calculate quality indicators under development. Question 11 reached a level of agreement above 0.80 (Fig 1) and a moderate agreement according to the kappa value (Fig 2), while question 21 and 23 showed levels of agreement below 0.80 (Fig 1) and fair agreement according to the kappa values (Fig 2).

In the previous validation study [13] the question about symptoms showed low validity. In this study, the item about dyspnoea occurrence (20e1) had level of agreement above 0.80, the items about pain, death rattle and nausea occurrence (20a1, 20b1, and 20c1) had levels of agreement between 0.70 and 0.79 and the items about anxiety and confusion occurrence (20d1 and 20f1) had levels of agreement below 0.70. Three symptom occurrence items had moderate agreement, two had fair agreement and one poor agreement according to the kappa values. None of the six questions about symptom relief could be included in the analysis.

Three ELQ items that showed levels of agreement below 0.80 in the previous validation study [13] now showed levels of agreement above 0.80: question 10 about ability to express will and take part in decisions concerning the content of medical care, question 11 about information about transition to end-of-life care (ITEOL) to patients and question 17 about ITEOL to next of kin. The items about ITEOL to patient and next of kin have been changed in the new version of the ELQ and now specify that only documented ITEOL should be reported with a “Yes” in the ELQ. The item about pain assessment (question 21) fell below 0.80 in both studies.

### Comparison between ELQ registrations in the SRPC and paper forms

Two problems were identified when comparing the paper forms and the ELQ registrations: sometimes questions had been left unanswered in the paper forms, and sometimes questions had been answered in a technically incorrect way. These cases were excluded from further analysis (Table 4). Ten ELQ items were follow-up questions only to be answered when certain answer alternatives were chosen in the previous question, and thus only some cases could be used for analysis of these questions (Table 4). Six items (number 13b, 14b, 20c2, 20d2, 20e2 and 20f2) were excluded from further analysis because less than 50 cases remained.

**Table 4:** Number of included and excluded cases for the ELQ items that did not include all 100 cases in the comparison between ELQ registrations in the SRPC and paper forms.

Question in ELQ where all 100 cases were not analysed	Valid cases (n)	Reason for cases missing		
		No answer in paper ELQ (n)	Technically incorrect answer in paper ELQ (n)	Follow-up question not to be answered because of the answer to the previous question (n)
4. Date of death	99	-	1	-
5. Date of admission	96	4	-	-
11. ITEOL*** to patient **	99	1	-	-
13a. Pressure ulcer at arrival to healthcare unit	99	1	-	-
13b. Documentation of pressure ulcer at arrival	8	-	-	92
14a. Pressure ulcer at death *	89	11	-	-
14b. Documented pressure ulcer at death	24	-	-	76
15a. Assessment of oral health	98	1	1	-
15b. Disorder noted during oral health assessment	82	1	-	17
15c. Documented assessment of oral health *	81	1	-	18
16. Company at moment of death	99	1	-	-
17. ITEOL* to next of kin	99	1	-	-
18. Next of kin offered a follow-up appointment	99	1	-	-
20a2 Pain relief	70	2	-	28
20b1 Death rattle	98	2	-	-
20b2 Death rattle relief	53	2	-	45
20c1 Nausea	99	1	-	-
20c2 Nausea relief	13	-	-	87
20d1 Anxiety	99	1	-	-
20d2 Anxiety relief	42	1	-	57
20e1 Dyspnoea	92	7	1	-
20e2 Dyspnoea relief	23	-	1	76
20f1 Confusion	98	2	-	-
20f2 Confusion relief	27	-	-	73
21. Pain assessment **	98	2	-	-
22. Severe pain	97	3	-	-
23. Assessment of symptoms other than pain **	96	4	-	-
24.1 “As needed” opioid against pain *	99	1	-	-
25. Last date for physician examination	99	1	-	-

* Items used to calculate quality indicators.

** Items used to calculate quality indicators under development.

*** ITEOL–information about transition to end-of-life care.

The level of agreement varied between 0.93 and 1.00 when comparing the ELQ registrations in the SRPC and the paper forms. The lowest levels of agreement were seen for question 5 about date of admission (0.93) and question 7 about cause of death (0.95), indicating some transcription problems since the paper forms were transferred to the web-based form by a secretary and not by the nurse or physician that had filled out the ELQ in paper form. The kappa values varied between 0.66 and 1.00. Two items showed good and 24 showed very good agreement according to the kappa values. The lowest kappa values were calculated for question 18 about whether next of kin were offered a follow-up appointment (0.66), question 24.3 about “as needed” drugs against nausea (0.80) and question 17 about ITEOL to next of kin (0.89).

## Discussion

This study provides information about the validity of the new, revised version of the ELQ from the SRPC. This is important since the ELQ is used to gather Swedish national data about end-of-life care that is used for research purposes and by Swedish authorities. The ELQ now contains more items with high levels of agreement between registrations in the SRPC and notes in the patients’ medical records compared to the previous version, but some validity problems remain.

The items about symptoms are important both for clinical use and for research purposes, and to further improve the validity of these items poses an important challenge for future SRPC development. Most items about symptom occurrence still have room for validity improvement. The items about symptom relief could not be analysed, which was partly caused by lack of documentation in the medical records. Symptom relief is central in palliative care, but the documentation practice is evidently lacking. Surprisingly, documentation of pain relief was lacking in more cases than documentation about relief of other symptoms. Based on the findings by Gholiha et al [19], there is reason to believe that the documentation of symptoms is lacking even more outside the specialised palliative care.

The symptom information in the SRPC is provided by healthcare professionals. When possible, patient generated data is preferable. Symptom rating by proxies (next of kin or healthcare professionals) has been shown to differ from the patient’s own rating [23,24]. For example, Broberger et al. showed in a study on lung cancer patients that both family caregivers and nurses generally rated symptom occurrence as greater than what the patients did [25].

The planned technical approach to future data gathering is to transfer data directly from the medical records into the register[12], which would require better structure for documenting end-of-life care in the patients’ regular medical records. This solution would avoid the risk of mistakes when completing a paper form of the ELQ and transferring it to the web based form and diminish the time consuming process of documenting in two systems, but does not solve the problem of lack of documentation or the possibility of wrong information in the medical records.

There are examples of quality registers covering specialised palliative care in Denmark and Australia [26,27], but we have not found documentation in the literature about other registers outside the SRPC that collect national data about patient outcome measurements outside specialised palliative care. The SRPC is a unique database containing information about important quality issues in end-of-life care and is an important source of knowledge about Swedish end-of-life care. The SRPC collects some “soft” data with an inherent complexity in assessment, where a moderate validity maybe has to be accepted, but at the same time implicating a restricted use of these items in research studies.

### Methodological considerations

Because the ELQ items differ in the construction throughout the ELQ it is probably not meaningful to compare the exact levels of agreement or kappa values between different ELQ items. The information documented in the medical records was used as gold standard, thus the criterion validity was examined [28]. The fact that the gold standard used to validate quality register data may be incorrect has been stressed for validity surveys of other quality registers [29]. Possible ways for future studies to get better information about the patients actual end-of-life care could be interviews with next of kin or observations.

It is likely that staff at the specialised palliative healthcare units in this study misinterpret the questions in the ELQ to a lesser extent than staff at healthcare units without palliative specialisation would do. The same type of study using data from healthcare units that do not have a palliative specialisation would examine the ELQ in a population more similar to the general population registered in the SRPC, but would probably not be feasible due to presumed lack of documentation in the medical records. We were not able to sufficiently examine all causes of death included in question 7 because of the predominance of patients with cancer in this study. The sample size of 100 patients was not large enough to fully explore the validity of some ELQ items where all 100 cases could not be used.

### Conclusion

Our study showed that the ELQ now contains more items with high levels of agreement between registrations in the SRPC and notes in the patients’ medical records compared to the previous version, but some validity problems remain. The revision of the ELQ has strengthened its use as a tool for gathering data about quality of end-of-life care, but validating issues around symptom assessment remains a challenge in our model of quality assessment.

## Supporting information

S1 Questionnaire End-of-life questionnaire in use from April 2012 (English version). (DOCX)

S2 Questionnaire End-of-life questionnaire in use between May 2007 and December 2010 (English version). (DOCX)
