# Supplementary material for: Improved data validity in the Swedish Register of Palliative Care
Source: PLoS One. 2017 Oct 19;12(10):e0186804. doi: 10.1371/journal.pone.0186804 (PMC5648220; doi:10.1371/journal.pone.0186804)
Supplement: S1 Questionnaire — (DOCX) [file pone.0186804.s001.docx]

**APPENDIX 1**

**End-of-life questionnaire in use from April 2012 (English version)**

1. Unit identification code_______________[is automatically answered in the digital questionnaire]

2. Personal identity number of the deceased person (12 numbers)_______________

3. First and last name of the deceased person_______________

4. Date of death (year/month/day)_______________

5. Date (year/month/day) when the person was admitted to the unit where the death occurred (for home care, please state the date when home care was initiated)_______________

6. The place of death is best described as

□ Nursing home—permanent stay
□ Nursing home—short-term stay
□ Hospital ward (not hospice/palliative inpatient care)
□ Hospice/palliative inpatient care
□ Own home with support from specialised home-care team
□ Own home with support from general home-care team
□ Other, specify_______________

7. Disease/basic state that caused the death (more than one answer is possible):

□ Cancer
□ Cardiovascular disease
□ Respiratory disease
□ Dementia
□ Stroke
□ Other neurological disease
□ Diabetes
□ State after fracture
□ Multimorbidity
□ Other, namely:_______________

8. Will there be a postmortem examination of the deceased?

□ Yes, forensic □ Yes, regular clinical □ No

If the answer is Yes, forensic, only answer questions 28–30. If the answer is Yes, regular clinical or No, go to question 9.

9. Based on the disease trajectory, was the death expected?

□ Yes □ No □ Don’t know

If the answer is Yes or Don’t know, answer all the following questions. If the answer is No, answer only questions 14, 16, 18, 28–30.

10. How long before death did the person lose the ability to express his/her will and take part in decisions concerning the content of medical care?

□ Retained ability until end of life □ Hour/hours □ Day/days
□ Week/weeks □ Month or more □ Don't know

11a. Do the medical records include a documented decision by the physician responsible to shift treatment/care to end-of-life care?

□ Yes, in free text □ Yes, as a classification code □ No □ Don’t know

11b. Did the person receive information about the transition to end-of-life care, i.e. an individually tailored and informed conversation with a physician that is documented in the medical records about being in the final stage of life and about care being focused on quality of life and symptom relief?

□ Yes □ No □ Don’t know

12. Was the place of death in line with the person’s last stated wishes?

□ Yes □ No □ Don’t know

13a. Did the person have pressure ulcers upon arrival at your unit (specify highest grade occurring)?

□ Yes, grade 1 □ Yes, grade 2 □ Yes, grade 3 □ Yes, grade 4 □ No □ Don’t know

If the answer is Yes (grade 1–4), answer question 13b. If the answer is No or Don’t know, skip to question 14a.

13b. Were the pressure ulcers documented?

□ Yes □ No □ Don’t know

14a. Did the person die with pressure ulcers (specify highest grade occurring)?

□ Yes, grade 1 □ Yes, grade 2 □ Yes, grade 3 □ Yes, grade 4 □ No □ Don’t know

If the answer is Yes (grade 1–4), answer question 14b. If the answer is No or Don’t know, skip to question 15a.

14b. Were the pressure ulcers documented?

□ Yes □ No □ Don’t know

15a. Was the person’s oral health assessed during the last week of life?

□ Yes □ No □ Don’t know

If the answer is Yes, answer question 15b. If the answer is No or Don’t know, skip to question 16.

15b. Was any disorder noted during assessment?

□ Yes □ No □ Don’t know

If the answer is Yes or No, answer question 15c. If the answer is Don’t know, skip to question 16.

15c. Was the assessment of oral health documented?

□ Yes □ No □ Don’t know

16. Was anyone present at the time of death?

□ Yes, close friend(s) or relative(s) □ Yes, close friend(s)/relative(s) and staff
□ Yes, staff □ No □ Don’t know

17. Did the person’s close friend(s)/relative(s) receive information about transition to end-of-life care, i.e. an individually tailored and informed conversation with a physician that is documented in the medical records about being in the final stage of life and about care being focused on quality of life and symptom relief?

□ Yes □ No □ Don’t know □ Had no close friend(s)/relative(s)

If the answer is Yes, No or Don’t know, go to question 18. If the answer is Had no close friend(s)/relative(s), skip to question 19.

18. Was/were the person’s close friend(s)/relative(s) offered a follow-up talk within 1–2 months of the death?

□ Yes □ No □ Don’t know

19. Did the person receive parenteral fluids/nutrition or enteral-tube feeding during the last 24 hours of life?

□ Yes □ No □ Don’t know

20. Did the person display any of the following symptoms (20a–f) at any time during the last week of life?

20a. Pain □ Yes □ No □ Don’t know

If the answer is Yes, answer the following question. If the answer is No or Don’t know, skip to question 20b.

Pain was relieved: □ Completely □ Partially □ Not at all

20b. Death rattle □ Yes □ No □ Don’t know

If the answer is Yes, answer the following question. If the answer is No or Don’t know, skip to question 20c.

Death rattle was relieved: □ Completely □ Partially □ Not at all

20c. Nausea □ Yes □ No □ Don’t know

If the answer is Yes, answer the following question. If the answer is No or Don’t know, skip to question 20d.

Nausea was relieved: □ Completely □ Partially □ Not at all

20d. Anxiety □ Yes □ No □ Don’t know

If the answer is Yes, answer the following question. If the answer is No or Don’t know, skip to question 20e.

Anxiety was relieved: □ Completely □ Partially □ Not at all

20e. Dyspnoea □ Yes □ No □ Don’t know

If the answer is Yes, answer the following question. If the answer is No or Don’t know, skip to question 20f.

Dyspnoea was relieved: □ Completely □ Partially □ Not at all

20f. Confusion □ Yes □ No □ Don’t know

If the answer is Yes, answer the following question. If the answer is No or Don’t know, skip to question 21.

Confusion was relieved: □ Completely □ Partially □ Not at all

21. Was the person’s pain assessed at any documented time during the last week of life using VAS, NRS or another validated pain-assessment tool?

□ Yes □ No □ Don’t know

22. Did the person experience severe pain at any time during the last week of life (e.g. VAS/NRS > 6 or severe pain according to another validated pain-assessment tool)?

□ Yes □ No □ Don’t know

23. Were the person’s other symptoms assessed at any time during the last week of life using VAS, NRS or another validated symptom-assessment tool?

□ Yes □ No □ Don’t know

24. Was there an individual prescription of injectable PRN drugs on the drug list before death?

Opioids against pain □ Yes □ No □ Don’t know
Drugs against death rattle □ Yes □ No □ Don’t know
Drugs against nausea □ Yes □ No □ Don’t know
Drugs against anxiety □ Yes □ No □ Don’t know

25. How long before death was the person last examined by a physician?

□ Day/days □ Week/weeks □ Month or more □ Don’t know

26. Were specialists outside the team/ward consulted concerning the person’s symptom relief during the last week of life (more than one answer option is possible)?

□ Yes, pain clinic □ Yes, palliative care team □ Yes, other hospital unit
□ Yes, social worker/physiotherapist/ occupational therapist/dietician
□ Yes, spiritual counsellor □ No □ Don’t know

27. How satisfied is the team with the care delivered to the person during the last week of life?

□ 1 = Not at all □ 2 □ 3 □ 4 □ 5 = Completely

28. Date (year/month/day) of completing the questionnaire_______________

29. The questionnaire was completed by:

□ A single employee □ Staff jointly

30. Name of registrant_______________

□ Physician □ Nurse □ Other staff

E-mail address_______________
