# Supplementary material for: Improved data validity in the Swedish Register of Palliative Care
Source: PLoS One. 2017 Oct 19;12(10):e0186804. doi: 10.1371/journal.pone.0186804 (PMC5648220; doi:10.1371/journal.pone.0186804)
Supplement: S2 Questionnaire — (DOCX) [file pone.0186804.s002.docx]

**APPENDIX 2**

**End-of-life questionnaire in use between May 2007 and December 2010 (English version)**

1. Unit code_______________ [received at registration of participation through the website www.palliativ.se]

2. Social insurance identification number_______________

3. Name of the deceased_______________(first name and surname used)

4. Area code_______________

5. Sex □ Male □ Female

6. Date of admission to the unit where death occurred (for primary care/home care = “active home care”)_______________

7. Date of death_______________

8. The place of death is best described as a

□ Nursing home
□ Short-term care home
□ Hospital ward—not palliative
□ Hospice/palliative hospital ward
□ Own home, with support from specialised palliative home care
□ Own home, with support from basic home care
□ Other_______________

9. Main disease that caused death:

□ Cancer
□ Heart disease
□ Lung disease
□ Dementia
□ Stroke
□ Other neurological disease
□ Infection
□ Diabetes
□ Other, namely_______________

10. Will a forensic autopsy be performed?

□ Yes □ No

If the answer to question 10 is yes, then the questionnaire is completed. If death was caused by disease, please also answer the following questions.

11. According to the deceased’s medical history, death was

□ Expected □ Not expected □ Don’t know

12. Which date, closest before death, did a doctor visit/examine the patient/person receiving care?_______________

13. Has an informing conversation with the patient about impending death taken place, during the last period in life?

□ No □ Yes, by a doctor □ Yes, by a nurse
□ Yes, by both a doctor and a nurse □ Don’t know

14. Has an informing conversation with the patient’s next of kin about the impending death of the patient/person receiving care taken place, during the last period in life?

□ No □ Yes, by a doctor □ Yes, by a nurse
□ Yes, by both a doctor and a nurse □ Don’t know

15. How long before death did the patient/person receiving care lose his/her ability of self-determination?

□ Hours □ Days □ Weeks □ Months □ Years □ Don’t know

16. Has a VAS or NRS scale (0–10) been used for evaluation of pain during the last week of the patient’s life?

□ Yes □ No □ The patient cannot participate □ Don’t know

17. Mark the symptom(s) that was/were not fully alleviated during the last week of life.

□ Shortness of breath □ Death rattle □ Other_______________
□ Confusion □ Pain □ No distressing symptoms
□ Nausea □ Anxiety □ Don’t know

18. Has special competence outside the team/ward been consulted regarding the patient’s not completely alleviated symptoms?

□ No □ Yes, profession/speciality_______________

19. Did the person receiving care/patient have pressure ulcers in the last week of life?

□ Grade 1 □ Grade 2 □ Grade 3 □ Grade 4 □ No □ Don’t know

20. Was medication prescribed for use as needed in the form of injections, at least one day before death, for

Pain □ Yes □ No
Death rattle □ Yes □ No
Nausea □ Yes □ No
Anxiety □ Yes □ No

21. Who was present at the moment of death?

□ Staff □ Next of kin □ Staff and next of kin □ No one

22. Did the place of death correspond with the person receiving care’s/patient’s latest spoken wish?

□ Yes □ No □ Don’t know

23. In how many other places (e.g. home, different wards, nursing home, short-term care home) than the place of death was the person receiving care/patient cared for during the last 2 weeks of life?

□ 0 □ 1 □ 2 □ 3 □ >3 □ Don’t know

24. Have the next of kin had or will they be offered a follow-up appointment some time after death?

□ Yes □ No □ Don’t know

25. Are you content with the end-of- life care provided for the person receiving care/patient?

1 2 3 4 5
□ □ □ □ □
Not at all Completely

26. Date the questions were answered_______________

27. Completed by_______________(name)

□ Doctor □ Nurse E-mail address_______________
